# Effect of inspiratory muscle training in children with asthma: a systematic review and meta-analysis of randomized controlled trials

**DOI:** 10.3389/fped.2024.1367710

**Published:** 2024-03-18

**Authors:** Yuping Xiang, Tianhui Luo, Xinyang Chen, Huanhuan Zhang, Ling Zeng

**Affiliations:** Department of Critical Care Medicine, West China Hospital, Sichuan University/West China School of Nursing, Sichuan University, Chengdu, Sichuan, China

**Keywords:** inspiratory muscle training, children, asthma, maximum inspiratory pressure, systematic review

## Abstract

**Background:**

Asthma is a common chronic respiratory disease in children. Alongside pharmacological interventions, inspiratory muscle training (IMT) emerges as a complementary therapeutic approach for asthma management. However, the extent of its efficacy in pediatric populations remains uncertain when compared to its benefits in adults. This systematic review aims to evaluate the effectiveness of IMT with threshold loading in children with asthma.

**Methods:**

Randomized controlled trials (RCTs) evaluating the efficacy of inspiratory muscle training in pediatric asthma patients were identified through June 2023 across various literature databases, including PubMed, Embase, Cochrane Central Register of Controlled Trials (CENTRAL), Cumulative Index to Nursing and Allied Health Literature (CINAL), Web of Science, China Knowledge Resource Integrated Database (CNKI), Wei Pu Database, Wan Fang Database, and Chinese Biomedical Database (CBM). These trials compared inspiratory muscle training against sham inspiratory muscle training and conventional care. Eligible studies were assessed in terms of risk of bias and quality of evidence. Where feasible, data were pooled and subjected to meta-analysis, with results reported as mean differences (MDs) and 95% confidence intervals (CIs).

**Results:**

Six trials involving 333 patients were included in the analysis. IMT demonstrated significant improvements in maximum inspiratory pressure (MIP) (MD 25.36, 95% CI 2.47–48.26, *P *= 0.03), maximum expiratory pressure (MEP) (MD 14.72, 95% CI 4.21–25.24, *P *= 0.006), forced vital capacity in percent predicted values [FVC(% pred)] (MD 3.90, 95% CI 1.86–5.93, *P *= 0.0002), forced expiratory volume in the first second in percent predicted values [FEV_1_(% pred)] (MD 4.96, 95% CI 2.60–7.32, *P *< 0.0001), ratio of forced expiratory volume in 1 s to forced vital capacity (FEV_1_/FVC) (MD 4.94, 95% CI 2.66–7.21, *P *< 0.0001), and asthma control test (ACT) (MD = 1.86, 95% CI: 0.96–2.75, *P *< 0.0001).

**Conclusions:**

Findings from randomized controlled trials indicate that inspiratory muscle training enhances respiratory muscle strength and pulmonary function in pediatric asthma patients.

**Systematic Review Registration:**

www.crd.york.ac.uk/prospero/display_record.php?ID=CRD42023449918, identifier: CRD42023449918.

## Introduction

Asthma poses a serious global health challenge affecting individuals across all age demographics. It is characterized by chronic airway inflammation and variable expiratory airflow limitation, resulting in recurrent wheezing, breathlessness, chest tightness, and coughing, especially during nocturnal and early morning hours (1). The 2019 Global Burden of Disease Collaboration estimated that asthma affected approximately 262 million people worldwide (2), while the 2016 Centers for Disease Control and Prevention reported a pediatric asthma incidence ranging from 9.6%–10.5% (3). Studies indicate that asthma accounts for approximately 14 million missed school days for school-age children annually (4), with healthcare expenditures amounting to billions of dollars (5). Hence, asthma is considered a public health challenge and a major global concern for governments and healthcare professionals.

The key objectives of asthma management, outlined by the Global Initiative for Asthma (GINA) guidelines, include symptom control, prevention of exacerbations, maintenance of near-normal lung function, mitigation of drug-induced side effects, and enabling children to engage in daily activities without hindrance (6). Presently, pharmacologic therapies represent the cornerstone of asthma treatment. However, long-term pharmacological interventions are associated with adverse effects (7); for instance, prolonged corticosteroid usage reduces inspiratory muscle function in patients with asthma (8, 9). Hence, there is a pressing need for safer and more efficacious interventions.

Increased airway resistance and hyperinflation in patients with asthma may lead to respiratory muscle dysfunction (10). Recently, respiratory and physical therapy designed to improve respiratory muscle strength and lung function in children and adults has gradually become a supplementary treatment for patients with asthma (11). Examples of supplementary treatments include physical training (11), breathing exercises (12), and respiratory muscle training (13). Among these, inspiratory muscle training (IMT) stands out as the most available and cost-effectivecost nonpharmacological intervention for supplementary treatment. Previous studies have demonstrated that IMT reduces respiratory muscle weakness and enhances respiratory pressure, exercise capacity, and quality of life (14, 15).

Silva et al. (16), 2013 systematically concluded that IMT significantly increased inspiratory muscle strength in adults with asthma. Lista-Paz et al. (17) reported that IMT can significantly increase maximum inspiratory pressure (MIP) in adults with asthma. While most recent systematic reviews and meta-analyses have predominantly focused on adults with asthma, several RCTs have been conducted on children with asthma (13, 18–22). Therefore, we systematically reviewed the available evidence from RCTs to assess the efficacy of inspiratory muscle training in strengthening the respiratory muscles in children with asthma.

## Methods

This systematic review and meta-analysis were conducted in accordance with the “Preferred Reporting Items for Systematic Reviews and Meta-Analyses” (PRISMA) guidelines (23). The protocol for this systematic review was registered in the International Prospective Register of Systematic Reviews (CRD42023449918).

### Literature search and study inclusion

We systematically searched the following electronic databases for randomized controlled trials indexed through June 2023: PubMed, Embase, Cochrane Central Register of Controlled Trials (CENTRAL), Cumulative Index to Nursing and Allied Health Literature (CINAL), Web of Science, China Knowledge Resource Integrated Database (CNKI), Wan Fang Database, Chinese Biomedical Database (CBM), and Wei Pu Database. The search strings contained the following MeSH and other terms (Supplementary Material S1): (asthma *OR* asthma* *OR* bronchial asthma *OR* wheez*) *AND* (breathing exercises *OR* breathing exercise* *OR* respiratory muscle training *OR* inspiratory muscle training *OR* inspiratory muscle train* *OR* inspiratory muscle strength *OR* threshold load *OR* threshold device *OR* IMT *OR* RMT). Search strings were adapted to each database as necessary. The reference lists of the relevant articles were manually searched to identify additional publications.

### Study selection

Studies were included in our review and meta-analysis if they met the following criteria: (1) they were randomized controlled trials involving children with asthma, regardless of its severity; (2) the intervention group received inspiratory muscle training, while the reference or control group received either sham inspiratory muscle training or usual care, defined as medication, education or traditional physical therapy; (3) data were reported for at least one outcome among maximum inspiratory pressure (MIP), maximal expiratory pressure (MEP), forced vital capacity (FVC), forced expiratory volume in 1 s (FEV_1_), ratio of forced expiratory volume in 1 s to forced vital capacity (FEV_1_/FVC), peak expiratory flow (PEF), quality of life, safety and asthma symptoms. Studies were excluded if the full text was unavailable. No language restrictions were applied to the eligible studies.

### Data extraction

Two authors independently extracted the relevant data, with a third author available to resolve any disagreements. The extracted data included characteristics of the trial, including the first author's name, title, and year of publication; participant demographics, such as age, sex, and sample size; details of inspiratory muscle training in the experimental arm and interventions in the control arm, including method, frequency, duration, and intensity; and outcomes. In cases where data on outcomes were unclear, we contacted corresponding authors to obtain missing information.

### Assessment of study quality

The risk of bias (RoB) in the included trials was assessed independently by two investigators using the Cochrane Risk of Bias 2 tool (24). Quality was evaluated across the following domains: randomized sequence generation, allocation concealment, blinding of participants, blinding of therapists, blinding of outcome assessors, incomplete outcome data, bias due to funding, selective reporting of outcomes, and other bias. The risk of bias for each tool item was judged as low, high, or unclear. Visualization of RoB 2 was performed using Robvis software. Any disagreements in quality assessment were resolved through discussion with a third investigator.

### Statistical analysis

We used Cochrane Review Manager 5 to combine outcomes when possible. For all continuous outcomes, we extracted the sample size, post-intervention means, and standard deviations (SDs), as well as the sample size and number of events. Mean difference (MD) served as the effect size when studies employed the same tool for outcome assessment. Alternatively, standard mean difference (SMD) was used as the effect size if different tools were employed. All effect sizes were reported with 95% confidence intervals (CI). A significance value of *P *< 0.05 was considered statistically significant. Heterogeneity within pooled data was assessed using the chi-square test, Cochran's Q test, and inconsistency *I*^2^ test. If the heterogeneity was no higher than 50%, meta-analysis was conducted on the pooled data for the entire sample (25); otherwise, sub-group meta-analysis was performed based on age and asthma controls.

## Results

### Study selection

**Figure 1:**
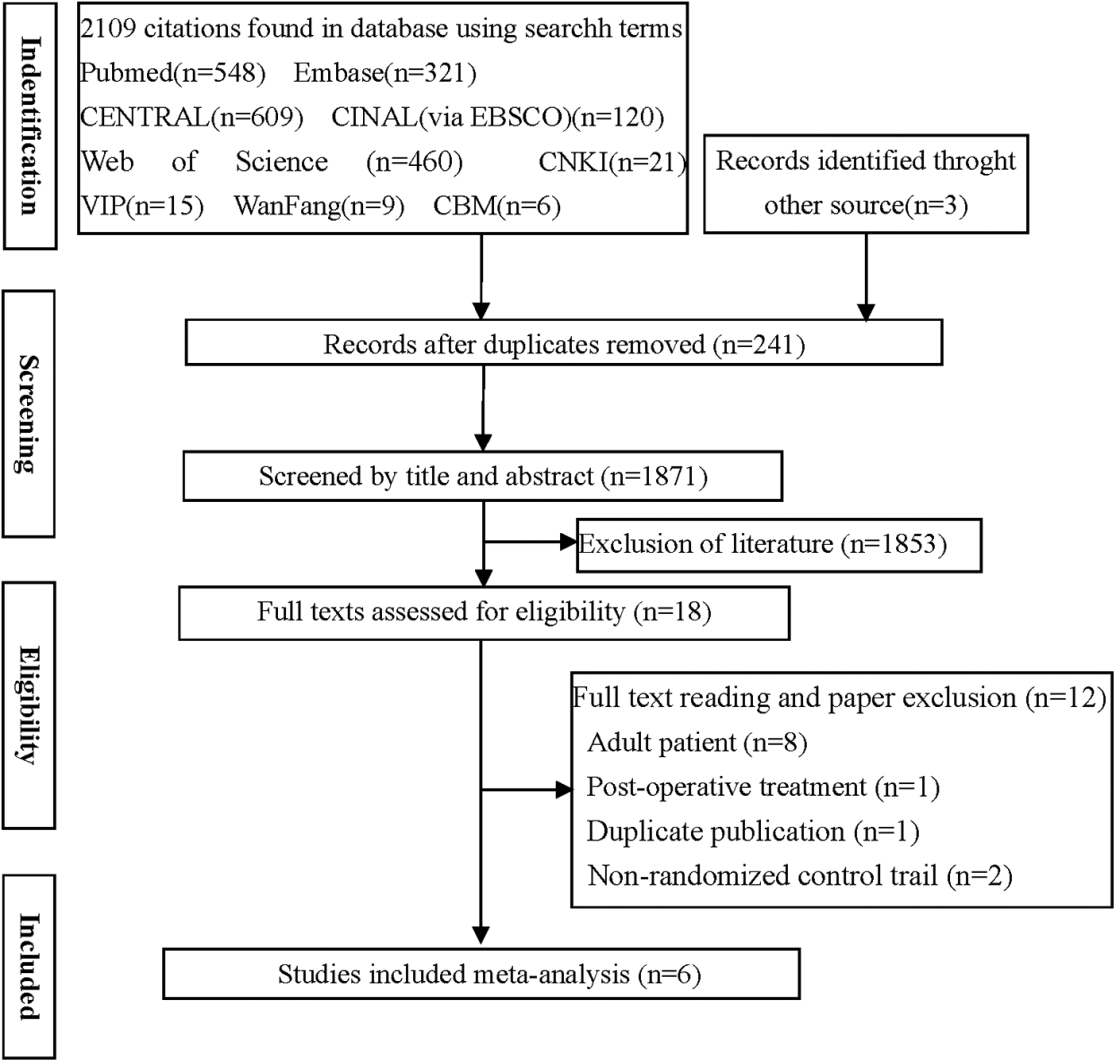
Flow diagram of the literature search.

Out of the 2,112 potentially relevant studies, 241 duplicates and 1,853 articles were excluded based on reading of titles and abstracts. The remaining 18 publications were read in full, following which six randomized controlled trials (13, 18–22) were retained for the final analysis (Figure 1, Table 1).

**Table 1:** Characteristics of included studies.

References	Country	Participants	Sample Size	Age (Years)	Type of intervention		Outcomes
			(IG/CG)		IG	CG	
Lima EV et al. (13)	Brazil	Clinical uncontrolled	50 (25/25)	(8–12)	IMT + medical visits and educational program	Medical visits and educational program	MIP:IG 109.9 (18.0) vs. CG 46.7 (4.1)
				IG:9.6 (1.2)			MEP:IG 82.0 (17.0) vs. CG 49.6 (5.5)
				CG:9.8 (1.2)			PEF:IG 312.0 (54.8) vs. CG 208.8 (44.2)
							Diurnal symptoms:IG 0 vs. CG 25, *P *< 0.001
							Nocturnal symptoms: IG 3 vs. CG 25, *P *< 0.001
							Impaired ability of daily living:IG 0 vs. CG 25, *P *< 0.001
							Asthma attacks:IG 2 vs. CG 22, *P *< 0.001
							Emergency room treatment:IG 3 vs. CG 8, *P *= 0.17
							Hospitalization:IG 3 vs. CG 3, *P *= 0.17
							Rescue bronchodilator use:IG 21 vs. CG 4, *P *< 0.001
David MMC et al. (22)	Brazil	Clinical controlled	42 (20/22)	(4–16)	IMT + respiratory exercises	Noninvasive ventilation CPAP(8 cm H_2_O)+respiratory exercises	MIP:IG 84.5 (29.6) vs. CG 59.7 (26.2)
				IG:11.0 (3.3)			MEP:IG 73.5 (27.1) vs. CG 52.0 (22.0)
				CG:9.0 (3.4)			FVC (% pred):IG 95.1 (13.5) vs. CG 97.8 (13.5)
							FEV1 (% pred):IG 82.2 (16.4) vs. CG 81.5 (15.7)
							FEV1/FVC:IG 85.2 (11.9) vs. CG 83.8 (10.6)
							FEF25–75 (% pred):IG 67.9 (26.1) vs. CG 68.3 (23.2)
							ACQ6: IG 0.33 (0–3) vs. 0.66 (0–2.5)
Elnaggar RK et al. (21)	NR	Clinical controlled	31 (16/15)	(12–16)	IMT + CRR	Sham IMT	MIP:IG 87.3 (8.1) vs. CG 78.5 (7.1)
				IG:14.6 (1.4)		(5% of pressure threshold)+CRR	MEP:IG 91.6 (7.3) vs. CG 82.4 (9.7)
				CG:13.9 (1.1)			FVC (% pred):IG 85.9 (3.7) vs. CG 79.7 (5.4)
							FEV1 (% pred):IG 79.6 (6.7) vs. CG 71.1 (5.6)
							FEV1/FVC:IG 86.9 (5.6) vs. CG 80.3 (4.9)
							ACT:IG 21.8 (1.3) vs. CG 19.7 (1.9)
Liu R et al. (20)	China	Clinical controlled	106 (51/55)	(4–12)	IMT + drug treatment	Drug treatment	FVC (% pred):IG 97.2 (10.6) vs. CG 93.4 (10.0)
				IG:6.3 (4.8, 7.7)			FEV1 (% pred):IG 101.8 (13.7) vs. CG 95.9 (12.7)
				CG:6.1 (4.7, 8.7)			PEF (% pred):IG 102.0 (12.7) vs. CG 90.2 (11.9)
							FEF25 (% pred):IG 93.7 (22.6) vs. CG 82.6 (24.4)
							FEF50 (% pred):IG 88.5 (21.6) vs. CG 83.8 (21.9)
							FEF75 (% pred):IG 87.0 (26.0) vs. CG 85.9 (22.7)
							Diurnal symptoms: IG 0.3 (0.1,0.4) vs. CG 0.4 (0.1,0.6)
							Nocturnal symptoms: IG 0.1 (0.0,0.3) vs. CG 0.3 (0.1,0.3)
							Pediatric Quality of Life Inventory version 4.0 generic core scales: IG 2114.1 (73.7) vs. CG 1,997.6 (95.1), *P *< 0.001
							Safety: IMT caused acute asthma attack was 0.8%
Gokcek O et al. (18)	Turkey	Clinical controlled	70 (35/35)	(8–17)	IMT	Drug treatment	MIP:IG 108.5 (24.7) vs. CG 81.9 (22.2)
				IG:11.4 (2.5)			MEP:IG 70.3 (14.0) vs. CG 60.3 (12.6)
				CG:12.5 (2.6)			FVC%:IG 97.9 (14.3) vs. CG 93.0 (13.2)
							FEV1%:IG 94.3 (13.7) vs. CG 88.2 (16.9)
							FEV1/FVC:IG 98.4 (9.6) vs. CG 89.8 (16.1)
							FEF25–75%:IG 87.9 (23.5) vs. CG 79.6 (24.2)
							CRP (mg/dl):IG 3.8 (1.9) vs. CG 3.4 (1.4), *P *= 0.31
Elnaggar RK et al. (19)	Saudi Arabia	Clinical controlled	34 (17/17)	(12–18)	IMT	Placebo training	MIP: IG 78.2 (6.4) vs. CG 74.5 (7.5)
				IG:15.12 (2.23)		no-load RMT	MEP: IG 76.9 (7.1) vs. CG 73.7 (5.6)
				CG:14.36 (1.97)			FVC (% pred): IG 73.4 (4.9) vs. 70.8 (5.8)
							FEV1 (% pred): IG 78.2 (6.9) vs. 75.8 (4.0)
							FEV1/FVC: IG 79.3 (5.4) vs. CG 76.2 (5.5)
							ACT: IG 19.2 (1.9) vs. CG 17.9 (2.1)

Values are expressed as mean ± standard deviation (SD) or median, ACQ6 expressed in median, minimum and maximum values. unless otherwise noted.

IG, intervention group; CG, control group; NR, not reported; IMT: inspiratory muscle training; MIP, maximal inspiratory pressure; MEP, maximal expiratory pressure; FVC, forced vital capacity; FEV_1_, forced expiratory volume in one second; FEV_1_/FVC, proportion of actual FEV1 to the full FVC; PEF, peak expiratory flow; ACT, asthma control test; FEF, forced expiratory flow; ACQ6, Asthma Control Questionnaire; CRR, conventional respiratory rehabilitation; CPAP, continuous positive airway pressure; RMT, respiratory muscle training; CRP, c-reactive protein; % pred, percent predicted values.

### Study characteristics

Details of the six included studies are presented in Table 1. One study was published in Chinese (20), while the others were published in English. These six studies included 333 children with asthma, with mean ages ranging from 6 to 15 years. Inspiratory muscle training utilized pressure-threshold loading devices in all studies (Table 2). The training sessions were conducted at 30%–55% of MIP, except in one study (20), which occurred 2–7 times per week, lasting 20–35 min, and spanning 5–12 weeks.

**Table 2:** Details of IMT training in included studies.

Study	Equipment	Intensity	Details of raining	Training site	Sessions			Total IMT duration
					Duration	No. per week	Supervised	
Lima EV et al. (13)	Threshold IMT (Respironics, Cedar Grove, NJ,USA)	40% of MIP	The first 20 min, Threshold IMT was used in 10 series of 60 s each, rest periods of 60 s, the final 5 min, training uninterruptedly	Home	25 min	2	NR	7 weeks
David MMC et al. (22)	Threshold IMT (Respironics,Cedar Grove, NJ, USA)	30% of MIP, increased by 10% after the first five sessions	Using a load of 30% of respiratory muscle strength for 30 min.	Hospital	30 min	2	NR	5 weeks
Elnaggar RK et al. (21)	Threshold-loading device (Respironics, Cedar Grove, NJ, USA)	40% of MIP, adjusted training load every week, applied 40% and 50% of MIP	The first 15 min, device used for diaphragmatic breathing, 15 breaths with 10 s rest intervals, the final 5 min, breathe through the device continuously	Hospital	20 min	3	Physical therapist	12 weeks
Liu R et al. (20)	Threshold IMT (LEVENTON S.A.U,Brail, 259–12,000)	NR	Guide the use of inspiratory muscle training device for all subjects and their families in outpatient clinics	Home	20–30 min	3	NR	12 weeks
Gokcek O et al. (18)	Threshold IMT	30% of MIP	Training session was performed every day with 10–15 repetitions with the breathing apparatus and a rest break of 5–10 s	NR	30 min	7	NR	6 weeks
Elnaggar RK et al. (19)	Threshold IMT (Threshold, Respironics, USA)	30% of MIP, increased by 5% every two weeks, until it reached 55% of MIP	6 sets of IMT succeeded by 6 sets of no-load training, total 12 sets. Each set consisted of 3 min training followed by 2 min for rest.	Hospital	3 5min	3	Physical therapist	12 weeks

IMT, inspiratory muscle training; MIP, maximum inspiratory pressure; NR, not reported.

### Methodological quality

**Figure 2:**
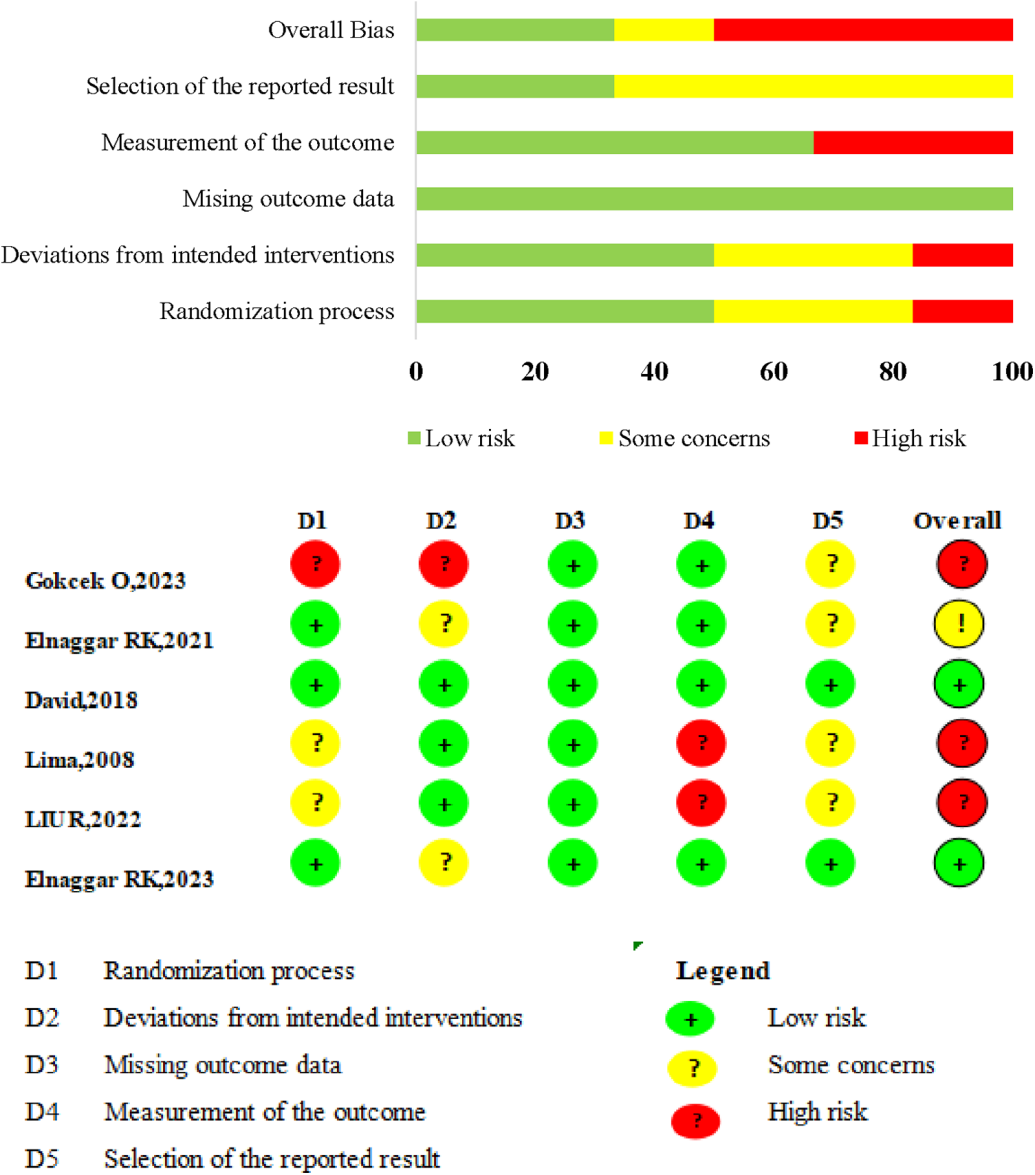
Risk of bias (ROB) summary. ROB 2.0 judgement according to domain and overall risk of bias for each study.

A summary and graph of the results of the RoB 2 tool are shown in Figure 2. Five studies featured at least one domain where the risk of bias was judged to be unclear, while three had a high risk of bias in at least one domain. Quality issues were noted in one study regarding subject randomization, another concerning subject allocation, and two in relation to outcome measurements.

### Outcome measures

#### Respiratory muscle strength (MIP and MEP)

**Figure 3:**
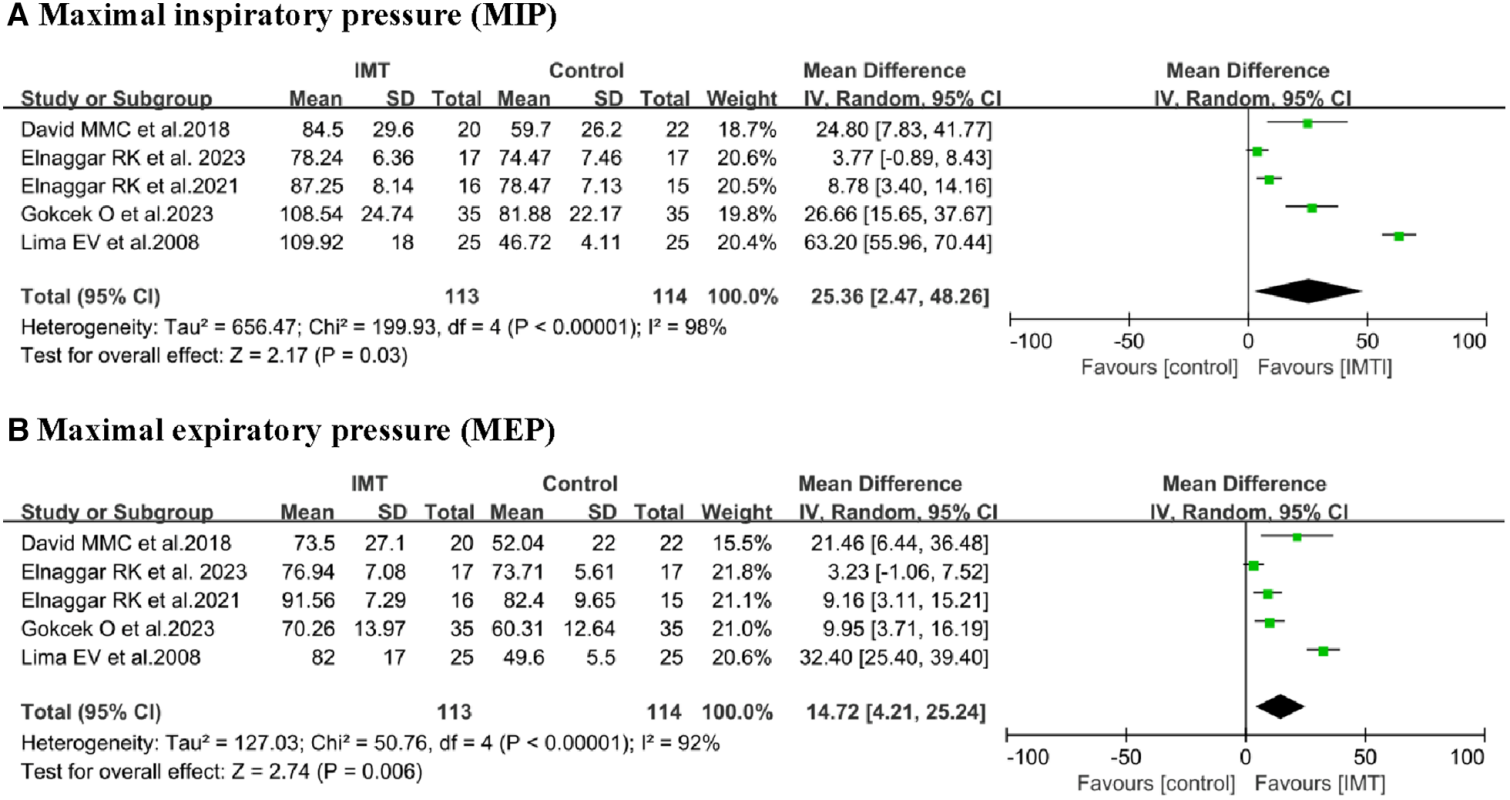
Comparison of MIP and MEP between inspiratory muscle training (IMT) and control groups.

*MIP:* Data from five studies (13, 18, 19, 21, 22) involving 227 children were extracted for meta-analysis to assess the effects of IMT on MIP. The training led to significant increase in MIP compared to control interventions (MD 25.36 cmH_2_O, 95% CI 2.47–48.26, *P *= 0.03; Figure 3). However, this outcome was associated with high heterogeneity (*I*^2^ = 98%). Subgroup analysis confirmed a significant difference when participants were stratified by age or asthma control (Table 3).

**Table 3:** Subgroup analyses of MIP and MEP.

Item	Included studies	Sample	Heterogeneity		MD (95% CI)	*P* value
			*I* ^2^	*P* value		
MIP(cmH_2_O)						
Age						
≤12 years	1 (13)	50	-	-	63.20 (55.96–70.44)	<0.00001
>12 years	2 (19, 21)	65	47%	0.17	5.92 (2.40–9.44)	0.001
Children and adolescents	2 (18, 22)	112	0%	0.86	26.11 (16.87–35.34)	<0.00001
Control of symptoms						
Uncontrolled	1 (13)	50	-	-	63.20 (55.96–70.44)	<0.00001
Controlled	4 (18, 19, 21, 22)	177	83%	0.0004	13.92 (4.45–23.40)	0.004
MEP(cmH_2_O)						
Age						
≤12 years	1 (13)	50	-	-	32.40 (25.40–39.40)	<0.00001
>12 years	2 (19, 21)	65	59%	0.12	5.80 (0.04–11.55)	0.05
Children and adolescents	2 (18, 22)	112	48%	0.17	11.65 (5.88–17.41)	<0.0001
Control of symptoms						
Uncontrolled	1 (13)	50	-	-	32.40 (25.40–39.40)	<0.00001
Controlled	4 (18, 19, 21, 22)	177	62%	0.05	8.58 (3.17–13.99)	0.002

MIP, maximal inspiratory pressure; MEP, maximal expiratory pressure.

*MEP:* Data from five studies (13, 18, 19, 21, 22) involving 227 children were extracted for meta-analysis to assess the effects of IMT on MEP. Due to the high level of homogeneity (*I*^2^ = 92%), a random-effects model was employed. The training led to significant improvement in MEP (MD 14.72 cmH2O, 95% CI: 4.21–25.24, *P *= 0.006; Figure 3). Subgroup analyses based on age and asthma control confirmed a significant difference (Table 3).

#### Pulmonary function

**Figure 4:**
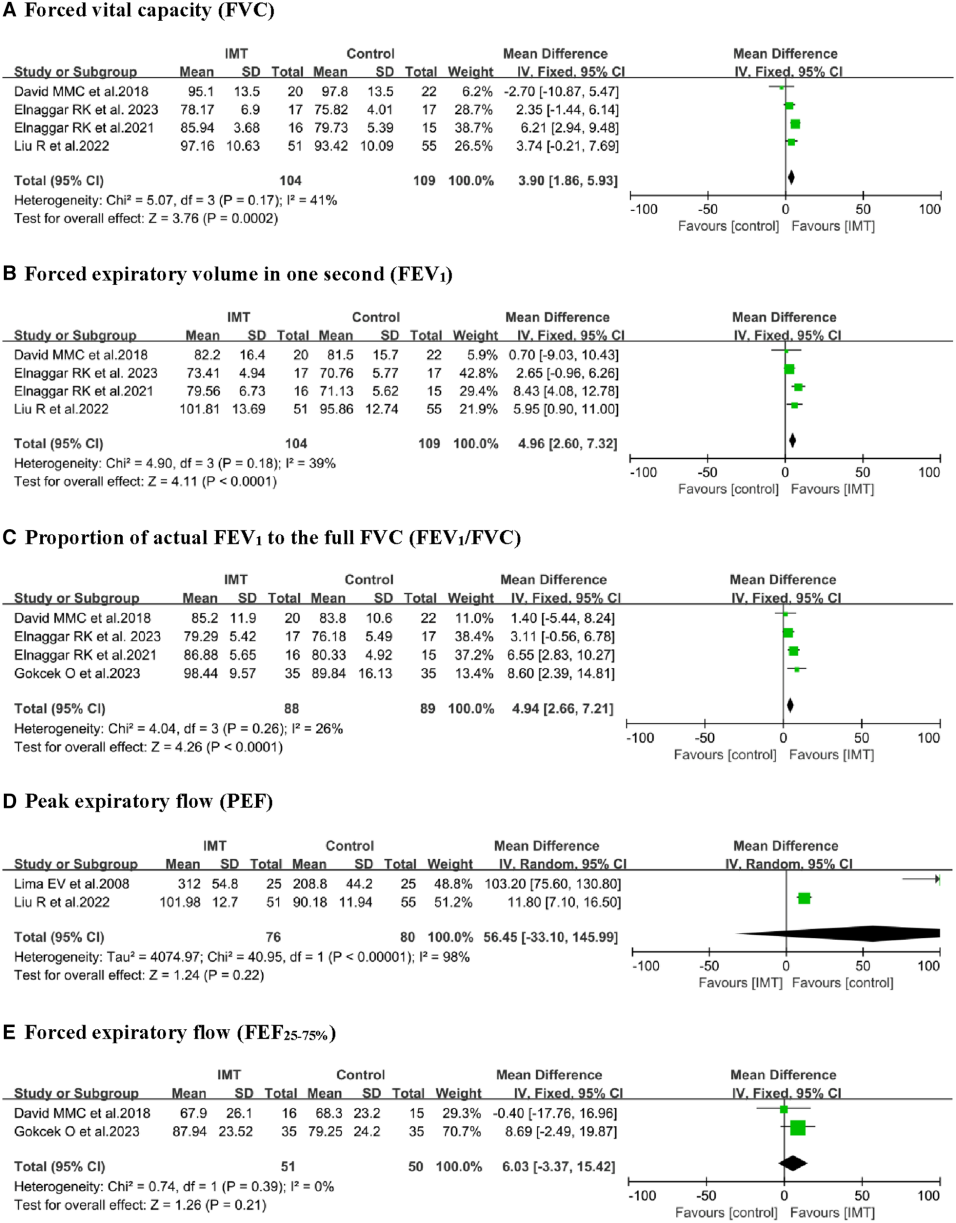
Forest plot of the effect of IMT on pulmonary function.

Five studies (18–22) reported the effect of IMT on FVC and FEV_1_, with four of them undergoing meta-analysis (19–22). A fixed-effect model was used to analyze and compare the IMT groups to control groups, where IMT was significantly associated with increased FVC (% predicted, MD 3.90, 95% CI: 1.86–5.93, *P *= 0.0002) and FEV_1_ (% predicted, MD 4.96, 95% CI: 2.60–7.32, *P *< 0.0001; Figure 4). Pooled analysis of FEV_1_/FVC revealed significant differences (MD 4.94, 95% CI: 2.66–7.21, *P *< 0.0001) between the IMT and control group. FVC, FEV_1_, and FEV_1_/FVC pooled estimates showed with low heterogeneity. Additionally, IMT did not significantly differ from control interventions in terms of peak expiratory flow in percent predicted values [PEF (% predicted)] or forced expiratory flow from 25% to 75% of vital capacity [FEF_25–75_ (% predicted)].

#### Asthma control test (ACT)

**Figure 5:**
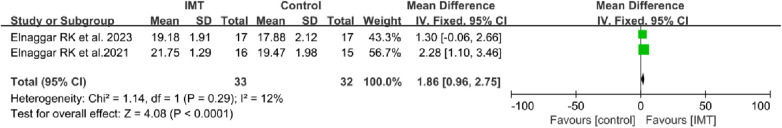
Forest plot of the effect of IMT on ACT.

Two of the included studies assessed the impact of IMT on the ACT (19, 21). The pooled analysis revealed a statistically difference in ACT scores between the IMT group and the control group (MD 1.86, 95% CI: 0.96–2.75, *P *< 0.0001; Figure 5).

#### Qualitative analysis of other outcomes

*Quality of life (QoL):* Liu (20). observed a significant increase in pediatric QoL inventory scores associated with IMT.

*Severity of asthma:* Lima et al. (13) linked asthma severity with significant improvements in diurnal and nocturnal asthma symptoms, daily living abilities, asthma attacks, and rescue use of bronchodilators.

*Safety:* Liu (20). reported that the incidence of acute asthma attacks due to IMT was 0.8%.

*Inflammatory markers:* Gokcek et al. (18) detected no significant differences in the levels of the inflammatory marker, C-reactive protein, between children who underwent inspiratory muscle training and those who received control interventions.

## Discussion

Our review and meta-analysis of six randomized controlled trials suggest that IMT can significantly improve MIP and MEP, as well as FVC, FEV_1,_ and FEV_1_/FVC of pulmonary function in children with asthma. We also observed significant differences in Asthma Control Test (ACT) between the IMT and control groups. In other words, available evidence supports the use of respiratory training in children with asthma. Additionally, we did not find extensive evidence either supporting or refuting the safety of IMT or its efficacy in improving quality of life or mitigating asthma severity. More evidence is needed to clarify the effect of IMT on quality of life, severity of asthma, and safety.

Asthma treatment comprises both pharmacological and non-pharmacological interventions. While medication therapy has long been used for asthma control, non-pharmacological approaches, such as educational programs (26), self-management (27), breathing exercises (12), and physical training (28), have been highlighted as adjuvant therapies for children undergoing pharmacological asthma treatment and are widely used worldwide. IMT is a therapeutic modality aimed at strengthening respiratory muscles and has some applications in children with asthma (13, 18–22).

To our knowledge, this is the first meta-analysis evaluating the efficacy of IMT in children with asthma. Our findings align with the results of IMT in asthmatic patients reported by Lista-Paz et al., 2023 (17), which included 11 studies, 10 with adults and only one study with children (13). These findings demonstrated a significant increase in MIP after IMT in adults with asthma, thereby reinforcing the relevance of the dose-response principle of training. Castilho et al. (12), published in 2020, investigated the effects of physical therapy on lung function in children with asthma across 18 studies; only two of these studies included IMT and conducted a qualitative analysis.

Training the respiratory muscles, particularly the inspiratory muscles, is recommended as part of the pulmonary rehabilitation program used in adults with asthma and chronic obstructive pulmonary disease (COPD) (16, 17, 29, 30). Research evidence indicates that IMT enhances inspiratory muscle strength and endurance, functional exercise capacity, and quality of life while decreasing dyspnea in COPD patients (29, 30). However, our meta-analysis was unable to determine the optimal duration of intervention or training intensity. A pressure threshold device was used for a session duration of 20–35 min. Regarding IMT intensity, 30%–55% of MIP was employed, and children reported that training with 40% of MIP (moderate intensity) was perceived more comfortable (21). Notably, only two studies reported training under the supervision of a physical therapist (19, 21). Additionally, no studies have reported adherence to IMT programs.

### Limitations

The findings of our review should be interpreted with caution, given several limitations of the included studies, primarily heterogeneity in participant age (4–18 years), variability in asthma control status, interventions utilized in the control arm, and inconsistencies in IMT intervention intensity. We found that IMT programs utilized external loads ranging from 30% to 55% of MIP. We attempted to address heterogeneity through subgroup analysis, which resulted in insufficient studies in some subgroups. The meta-analyses included no more than six trials, which precluded the assessment of publication bias using funnel plots. Due to the limited number of studies and small sample sizes, we recommend that future studies develop standardized protocols for IMT. Large randomized, placebo-controlled, double-blind trials would be a significant step forward in elucidating the effectiveness and safety of IMT in children and adolescents with asthma.

## Conclusion

Evidence from randomized controlled trials suggests that inspiratory muscle training can potentially strengthen respiratory muscles and improve pulmonary function in children with asthma. The efficacy and safety of such training should be explored in larger multicenter trials. Future research should explore whether inspiratory muscle training improves inspiratory muscle endurance, quality of life, asthma control, symptoms, severity, and safety.

## Data Availability

The raw data supporting the conclusions of this article will be made available by the authors, without undue reservation.
