# Supplementary material for: Effect of inspiratory muscle training in children with asthma: a systematic review and meta-analysis of randomized controlled trials
Source: Front Pediatr. 2024 Mar 18;12:1367710. doi: 10.3389/fped.2024.1367710 (PMC10982517; doi:10.3389/fped.2024.1367710)
Supplement: Supplementary file 1 [file Datasheet1.pdf]

1.Pubmed (548)

#1 "Breathing Exercises"[Mesh] 4,208

#2 (((((((((Breathing Exercise*[Title/Abstract]) OR (Breathing Train*[Title/Abstract])) OR (Respiratory Muscle Training[Title/Abstract])) OR (Inspiratory Muscle Training[Title/Abstract])) OR (Inspiratory Muscle Train*[Title/Abstract])) OR (Inspiratory Muscle Strength[Title/Abstract])) OR (threshold load[Title/Abstract])) OR (threshold device[Title/Abstract])) OR (respiratory train[Title/Abstract])) OR (IMT[Title/Abstract])) OR (RMT[Title/Abstract]) 14,950

#3 #1 OR #2 17,669

#4 "Asthma"[Mesh] 142,293

#5 ((Bronchial Asthma[Title/Abstract]) OR (asthma*[Title/Abstract])) OR (wheez*[Title/Abstract]) 187,883

#6 ("Asthma"[Mesh]) OR (((Bronchial Asthma[Title/Abstract]) OR (asthma*[Title/Abstract])) OR (wheez*[Title/Abstract])) 207,368

#7 #3 OR #6 548

Search: (("Breathing Exercises"[Mesh]) OR ((((((((((Breathing Exercise*[Title/Abstract]) OR (Breathing Train*[Title/Abstract])) OR (Respiratory Muscle Training[Title/Abstract])) OR (Inspiratory Muscle Training[Title/Abstract])) OR (Inspiratory Muscle Train*[Title/Abstract])) OR (Inspiratory Muscle Strength[Title/Abstract])) OR (threshold load[Title/Abstract])) OR (threshold device[Title/Abstract])) OR (respiratory train[Title/Abstract])) OR (IMT[Title/Abstract])) OR (RMT[Title/Abstract]))) AND (("Asthma"[Mesh]) OR (((Bronchial Asthma[Title/Abstract]) OR (asthma*[Title/Abstract])) OR (wheez*[Title/Abstract])))

2.Embase(321)

#1 'breathing exercise'/exp 10736

#2 'inspiratory muscle training':ti,ab,kw OR 'breathing exercise*':ti,ab,kw OR 'breathing train*':ti,ab,kw OR 'respiratory muscle training':ti,ab,kw OR 'inspiratory muscle train*':ti,ab,kw OR 'inspiratory muscle strength':ti,ab,kw OR 'threshold load':ti,ab,kw OR 'threshold device':ti,ab,kw OR 'respiratory train':ti,ab,kw OR imt:ti,ab,kw OR rmt:ti,ab,kw 23735

#3 #1 OR #2 31913

#4 'asthma'/exp 314662

#5 '':ti,ab,kw OR asthma*:ti,ab,kw OR wheez*:ti,ab,kw 272704

#6 #4 OR #5 362150

#7 'clinical trial':ti,ab,kw OR randomised:ti,ab,kw OR placebo:ti,ab,kw OR randomly:ti,ab,kw OR trial:ti,ab,kw OR groups:ti,ab,kw OR 'randomized controlled trial':ti,ab,kw 4837460

#8 #3 AND #6 AND #7 318

3.CENTRAL（609）

#1 (Breathing Exercises):ti,ab,kw OR (Breathing Exercise*):ti,ab,kw OR (Breathing Train*):ti,ab,kw OR (Respiratory Muscle Training):ti,ab,kw OR (Inspiratory Muscle Training):ti,ab,kw 10259

#2 (Inspiratory Muscle Train*):ti,ab,kw OR (Inspiratory Muscle Strength):ti,ab,kw OR (threshold load):ti,ab,kw OR (threshold device):ti,ab,kw OR (IMT):ti,ab,kw 5702

#3 #1 OR #2 14011

#4 MeSH descriptor: [Asthma] explode all trees 14992

#5 (Bronchial Asthma):ti,ab,kw OR (asthma*):ti,ab,kw OR (wheez*):ti,ab,kw 38629

#6 #4 OR #5 38629

#7 #3 AND #6 609

4.CINAL（120）

S1 AB Breathing Exercises OR AB Breathing Exercise* OR AB Breathing Train* OR AB Respiratory Muscle Training OR AB Inspiratory Muscle Training OR AB Inspiratory Muscle Train* OR AB Inspiratory Muscle Strength OR AB threshold load OR AB threshold device OR AB IMT OR AB RMT 5091

S2 AB Asthma OR AB Bronchial Asthma OR AB asthma* OR AB asthma* OR AB wheez* 31628

S3 S1 AND S2 120

5.web of science(460)

#1 (((((((((TS=(Breathing Exercises)) OR TS=(Breathing Exercise*)) OR TS=(Breathing Train*)) OR TS=(Respiratory Muscle Training)) OR TS=(Inspiratory Muscle Training)) OR TS=(Inspiratory Muscle Train*)) OR TS=(Inspiratory Muscle Strength)) OR TS=(threshold load)) OR TS=(threshold device)) OR TS=(IMT)) OR TS=(RMT) 85026

#2 (((TS=(Asthma)) OR TS=(Bronchial Asthma)) OR TS=(asthma*)) OR TS=(wheez*) 225756

#3 (((((TS=(clinical trial)) OR TS=(randomised)) OR TS=(randomly)) OR TS=(placebo)) OR TS=(trial)) OR TS=(groups) 6537052

#4 #3 AND #2 AND #1 460
